# Application of mendelian randomization to study the causal relationship between smoking and the risk of chronic obstructive pulmonary disease

**DOI:** 10.1371/journal.pone.0288783

**Published:** 2023-07-28

**Authors:** Yuenuo Huang, Xianchao Li, Wei Ye

**Affiliations:** Department of Medical Respiratory, Wenzhou TCM Hospital of Zhejiang Chinese Medical University, Zhejiang, China; Christian Medical College, INDIA

## Abstract

**Background:**

Smoking is a risk factor for chronic obstructive pulmonary disease (COPD). Few studies have assessed the causal relationship between smoking and COPD using Mendelian randomization.

**Methods:**

Exposure and outcome datasets were obtained from the IEU Open GWAS project (https://gwas.mrcieu.ac.uk/). The exposure data set includes smoking (ever smoke, smoking/smokers in household, exposure to tobacco smoke at home). The outcome data set includes COPD susceptibility and acute COPD admissions. The main methods of Mendelian randomization analysis are weighted median method and MR-Egger method. Heterogeneity and polymorphism analyses were performed to ensure the accuracy of the results.

**Resluts:**

ever smoke increased the risk of COPD prevalence, and ever smoke and smoking/smokers in household increased the risk of acute COPD admission. **Conclusion**

Therefore, we should enhance the management of nonpharmacological prescription of COPD to reduce the individual incidence.

## Introduction

Chronic obstructive pulmonary disease (COPD) is a type of chronic bronchitis and/or emphysema characterized by airflow obstruction, which can progress to pulmonary heart disease and respiratory failure [1]. COPD is a chronic progressive disease that may involve periods of acute exacerbation of respiratory symptoms beyond normal daily changes, which can be strictly referred to as acute exacerbations [2]. Acute exacerbations of COPD will have a negative impact on disease progression, resulting in decreased lung function, reduced quality of life and increased risk of death [3]. Therefore, early prevention for patients at risk of COPD is the cornerstone of management. Cigarette smoke not only contributes to the development of COPD, but also reduces the body’s ability to fight infections. COPD often leads to respiratory failure due to acute episodes of chronic infection or repeated infection [4]. In approximately 50% of nonsmoking COPD patients, quitting smoking is the most effective strategy for slowing COPD progression and reducing mortality [5].

Mendelian Randomization (MR) is a method for assessing etiological inferences in epidemiology. In non-experimental data, MR estimates the causal relationship between the exposure factor of interest and the outcome of interest by using genetic variation as an instrumental variable (Instrumental Variable, IV) [6]. The clear direction of cause and effect obtained by MR can avoid the influence of confounding factors and has a high reliability [6]. However, no studies have assessed the association between smoking and COPD susceptibility and hospital admission for acute episodes using Mendelian randomization analysis. We applied MR analysis to further investigate any association between genetic predisposition to smoking and COPD susceptibility and hospital admissions for acute episodes.

## Materials and methods

### Data sources

This study applied the genome-wide association study (GWAS) pooled data set to conduct a two-sample Mendelian randomization analysis. Through this analysis, the causal relationship between chronic obstructive pulmonary disease susceptibility, hospital admissions for acute episodes of chronic obstructive pulmonary disease (AECOPD) and smoking status was evaluated, and the sensitivity analyses was performed to verify the reliability of the results. The study was derived from the UK Biobank (https://gwas.mrcieu.ac.uk/), and data from the UK Biosample Repository were accessed via MR-Based. The outcome samples were classified into COPD susceptibility and AECOPD admissions. In addition, smoking status was classified into ever smoke, exposure to tobacco,smoker in household, and smoking data were also obtained from the UK Biobank.

### Instrumental variables

First, to satisfy the first MR hypothesis determination that SNPs must be strongly associated with the outcome variables (COPD susceptibility and AECOPD admission), SNPs significantly associated with episodes (children, adults) were selected at the genome-wide level (P<5×10–8,r2<0.001, genetic distance = 10 000 KB). If too few SNPs were included, the inclusion conditions could be appropriately changed as (P<5×10–5 to -14,r2<0.001,genetic distance = 10 000 KB). In order to ensure the second MR hypothesis that genetic variation is not associated with potential confounding factors, queries were performed in the Phenoscanner database to determine that the included SNPs were not associated with known confounding factors. Finally, we calculated the F statistics of the working variables to assess the extent of weak instrumental bias. Working varia `bles with F > 10 were retained to reduce the deviation caused by weak working variables. The formula for calculating F value was F = R2 (N-2)/1-R2.

### Mendelian analysis

In this study, MR Egger, Weighted median, Inverse variance weighted (IVW), Simple mode, and Weighted mode were used for two-sample Mendelian randomization analysis to explore the causal relationship between them. The traditional analysis method of Inverse variance weighting may be subject to invalid instrument bias or pleiotropy. Therefore, sensitivity analysis was conducted in this study to test the validity and robustness of the results. All statistical tests were performed using a two-tailed test. *P*< 0.05 was statistically significant.

## Results

### Selection of working variables

**Fig 1:**
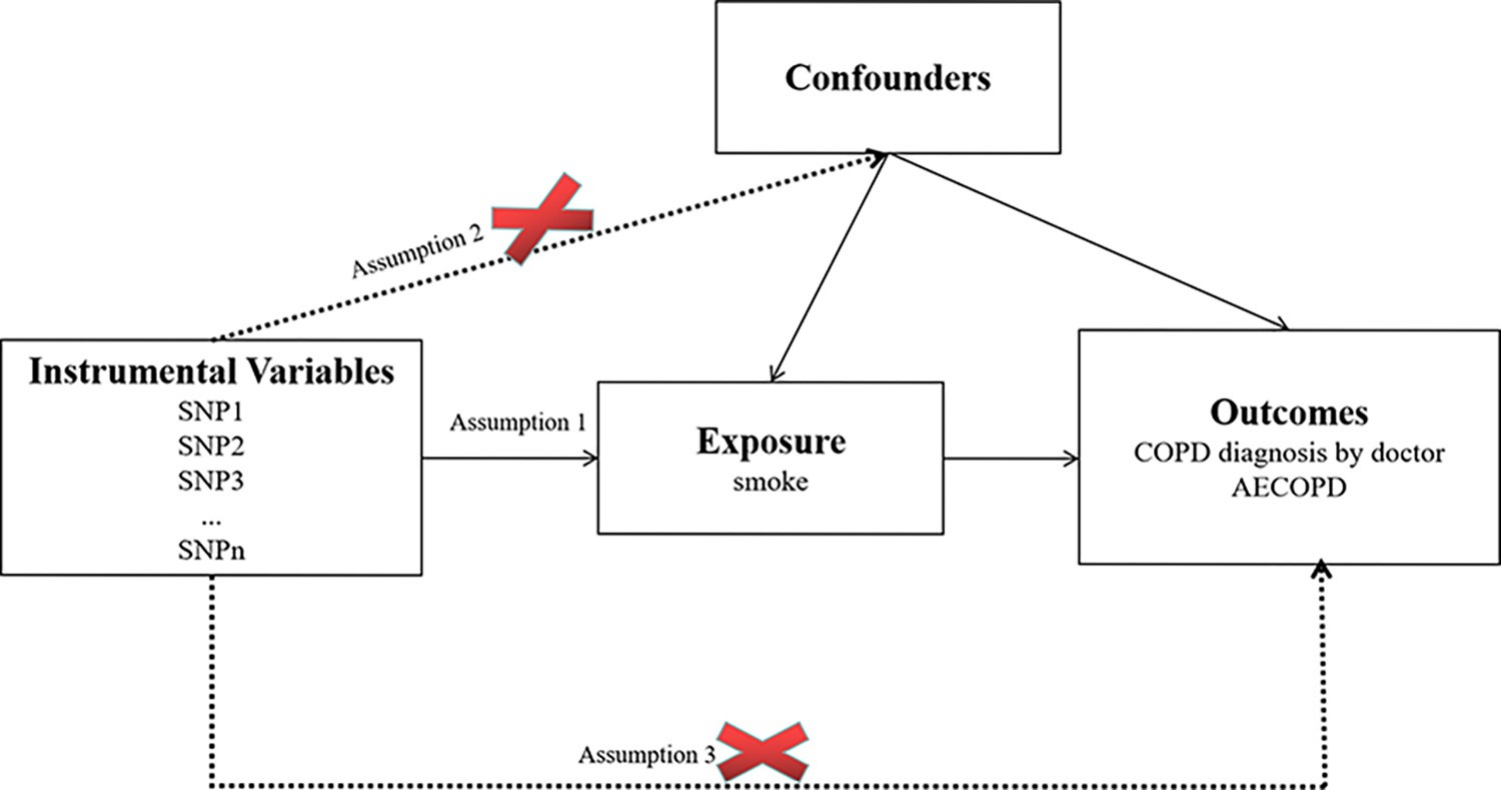
Flow chart of this study.

The SNPs of the working variables in our study are shown in S1 Table. The specific procedure is shown in Fig 1. The F-statistics of the SNPs included in our study were all greater than 10, indicating that the results of the study were not weakly biased, which confirmed that the results were reliable. The effect of each SNPs locus on COPD (susceptibility, admission due to acute exacerbation) was obtained by two-sample Mendelian randomization. See in S1A-S1F Fig in S7 File.

### Estimation of the causal relationship between smoking and COPD susceptibility

**Fig 2:**
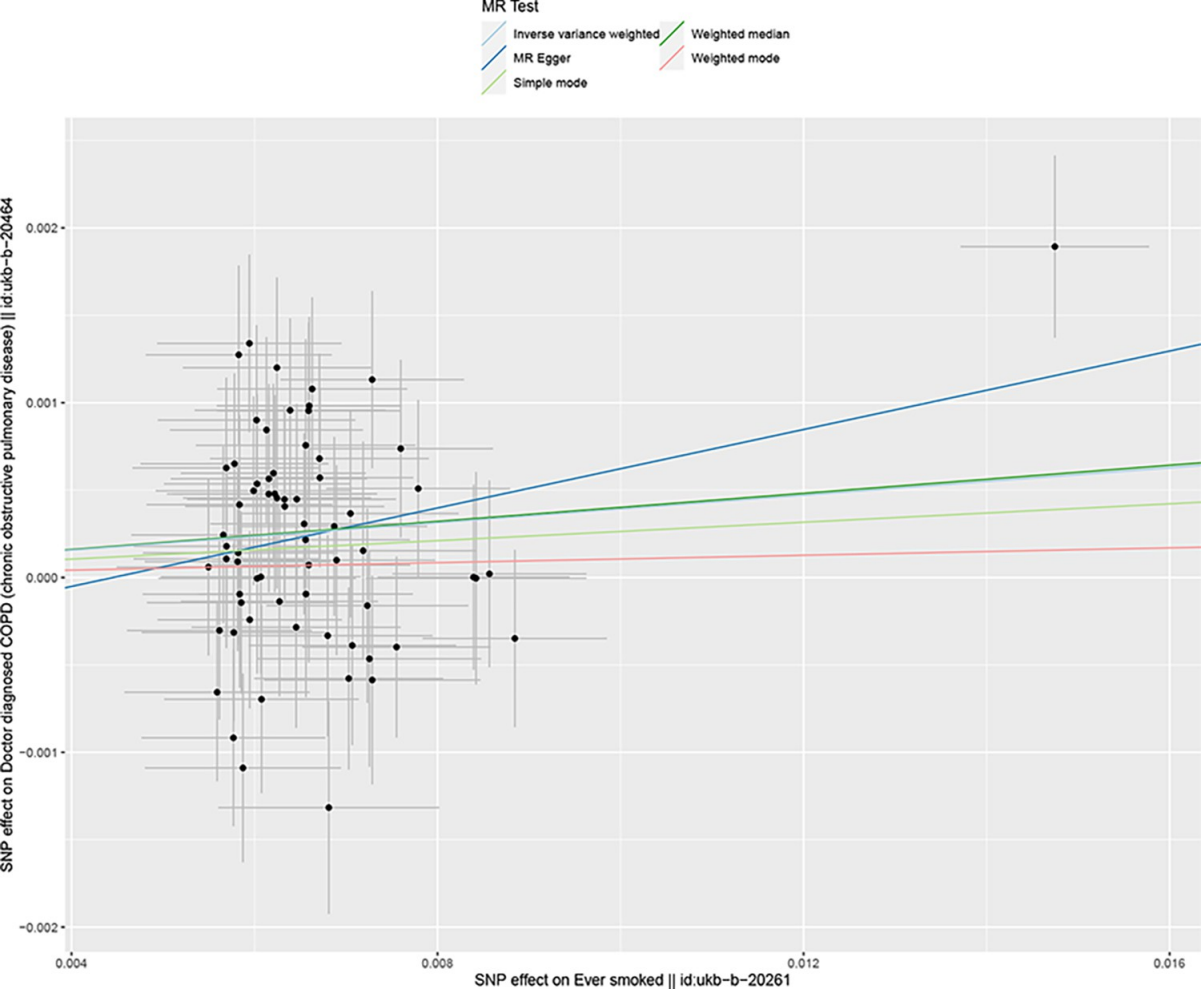
Scatter Chart of mendelian analysis between smoking and COPD susceptibility.

**Fig 3:**
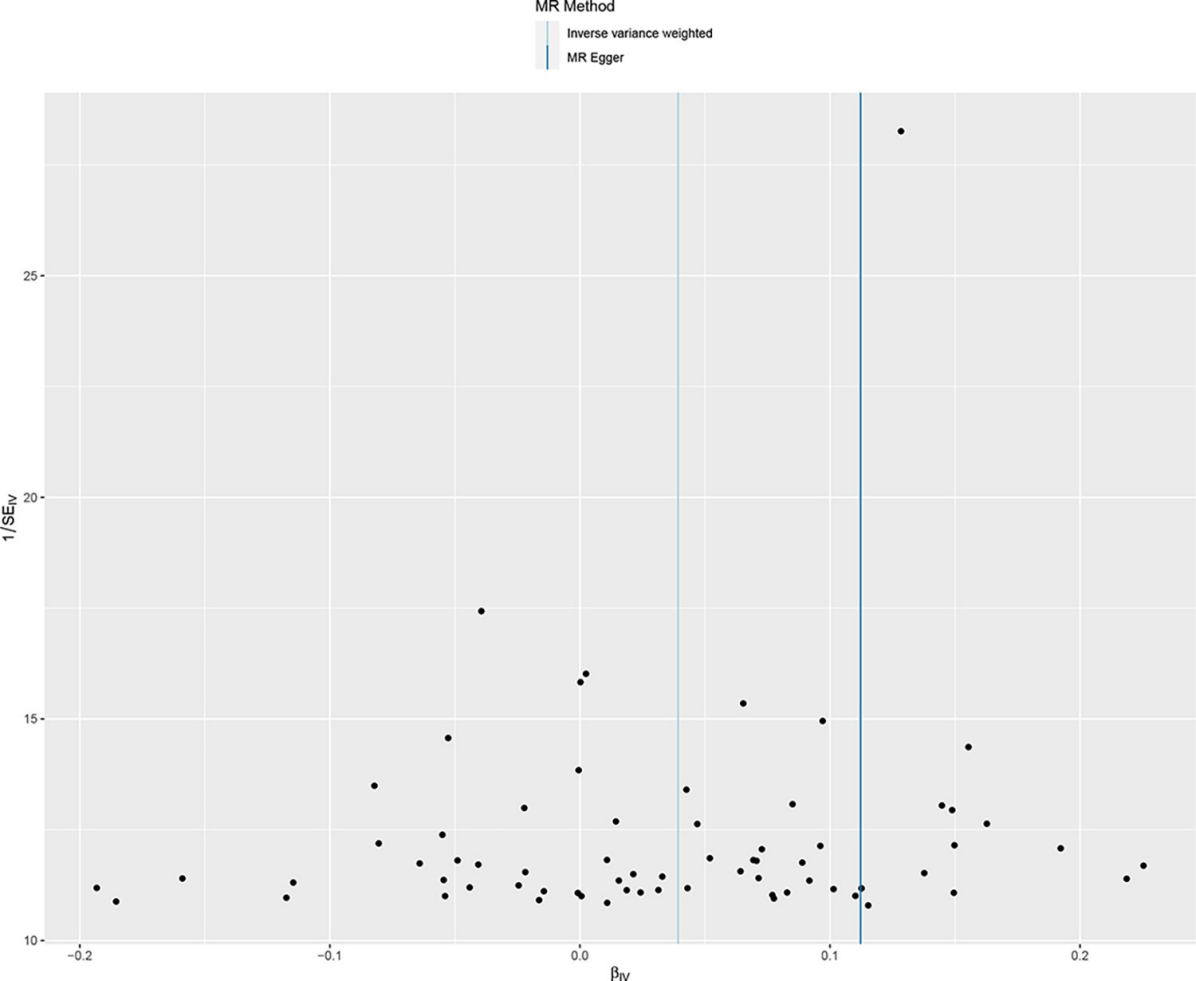
Funnel plot of mendelian analysis between smoking and COPD susceptibility.

**Fig 4:**
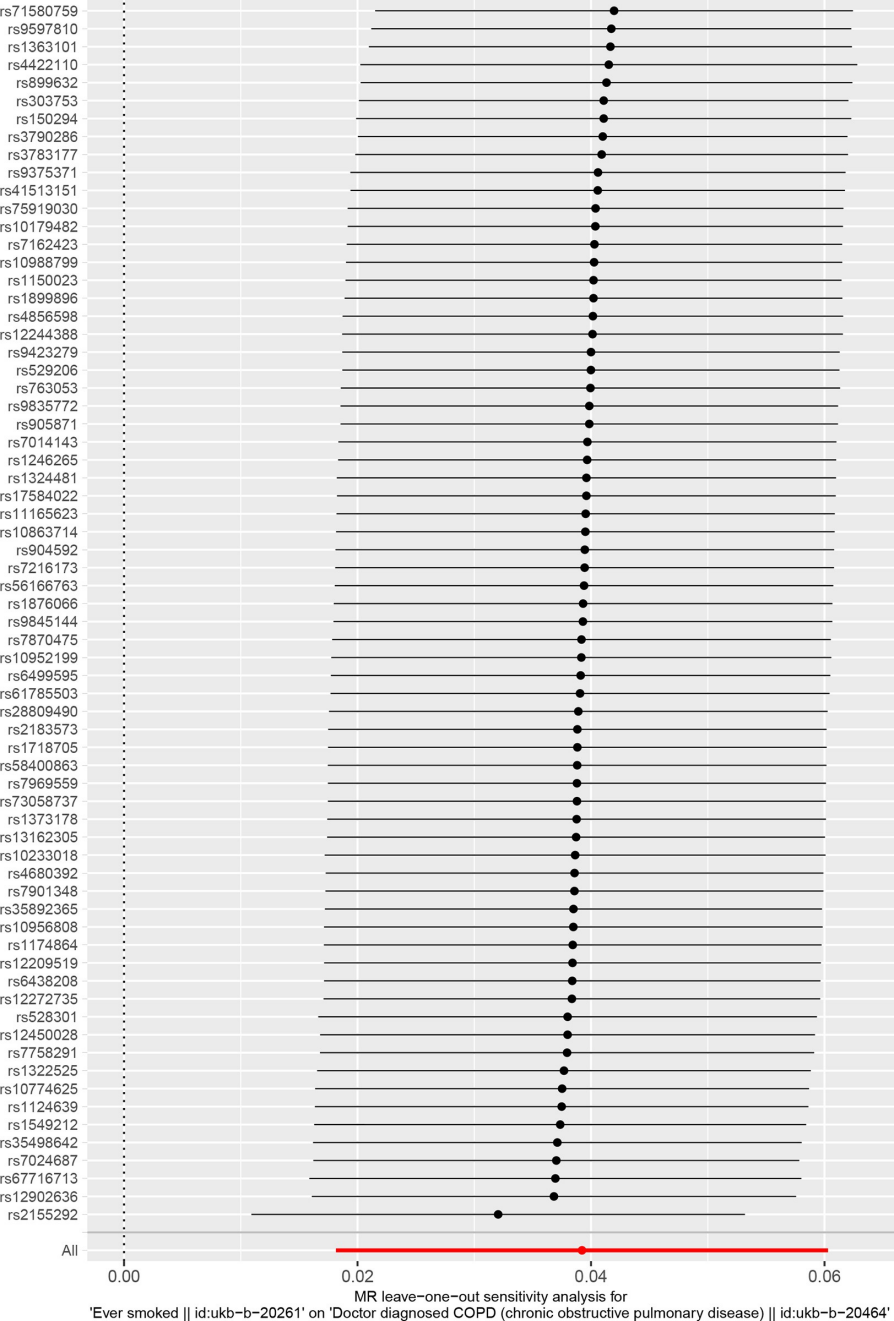
Leave one analysis of mendelian analysis between smoking and COPD susceptibility.

We included 68 SNPs according to the conditions ((P<5×10–8,r2<0.001,genetic distance = 10 000 KB). The IVM approach suggested that ever smoke was associated with COPD susceptibility and ever smoke increased COPD susceptibility. See in S2 Table. The scatter plot showed the estimated effect size of SNP for ever somked on COPD susceptibility (Fig 2). No significant heterogeneity was observed with the IVW test and MR-Egger test. See in S3 Table. The MR-Egger regression test did not support any evidence of directional variability. See in S3 Table. The MR-PRESSO test ensured the accuracy of the results. See in S3 Table. The symmetry of the funnel plot indicated the same results (Fig 3). In the omission analysis, we found no substantial change in the risk estimates of genetically predicted COPD after each SNP exclusion, suggesting that the potential driver SNPs are unlikely to bias the causality (Fig 4). The IVM approach showed that smoking/smokers in household, exposure to tobacco smoke at home were not associated with COPD susceptibility. See in S2 Table. The sensitivity analysis and heterogeneity analysis of smoking/smokers in household, exposure to tobacco smoke at home are shown in S3 Table.

### Estimation of the causal relationship between smoking and hospital admission for acute episodes of COPD

**Fig 5:**
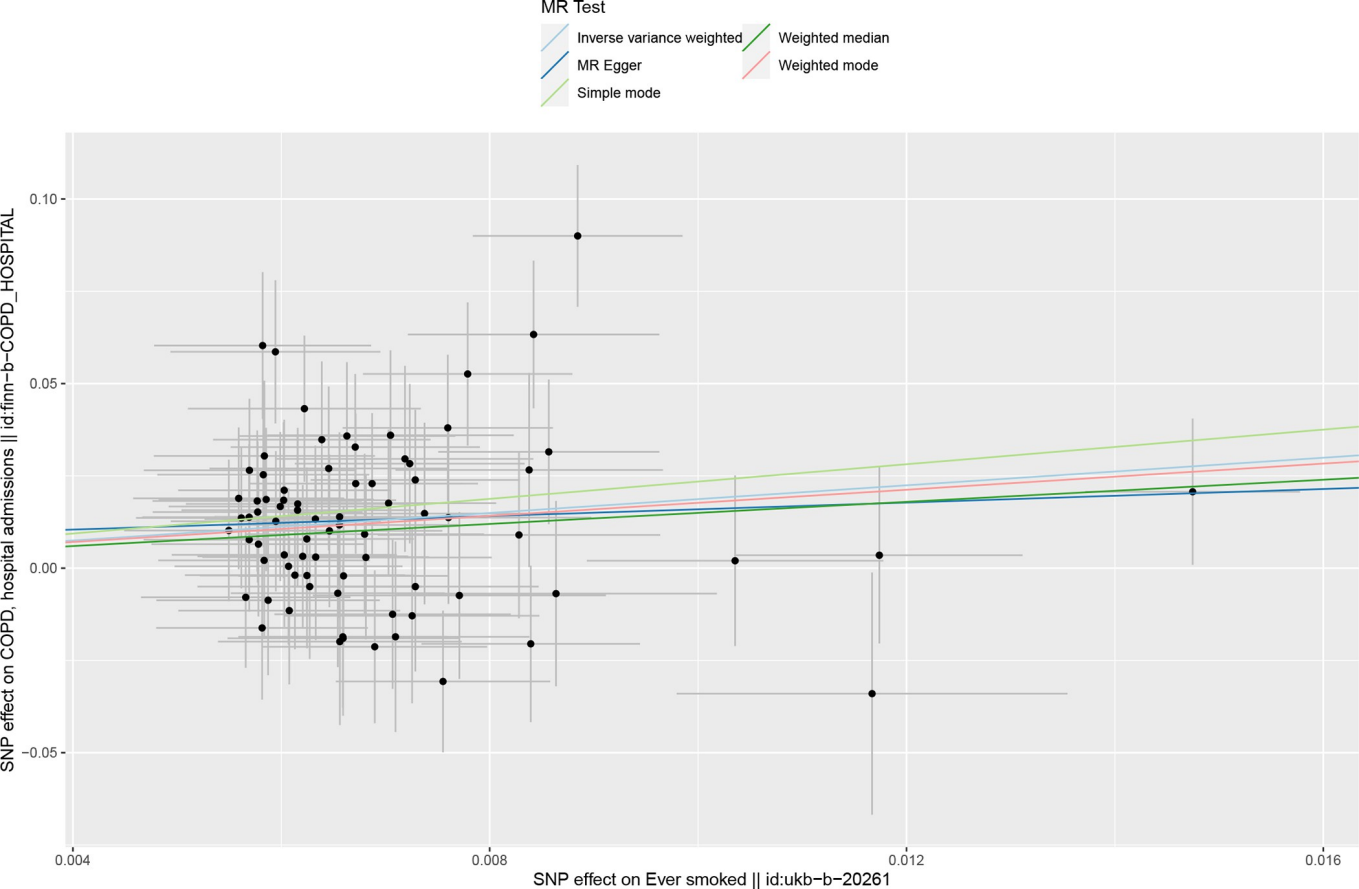
Scatter Chart of mendelian analysis between ever smoking andospital admission and acute episodes of COPD.

**Fig 6:**
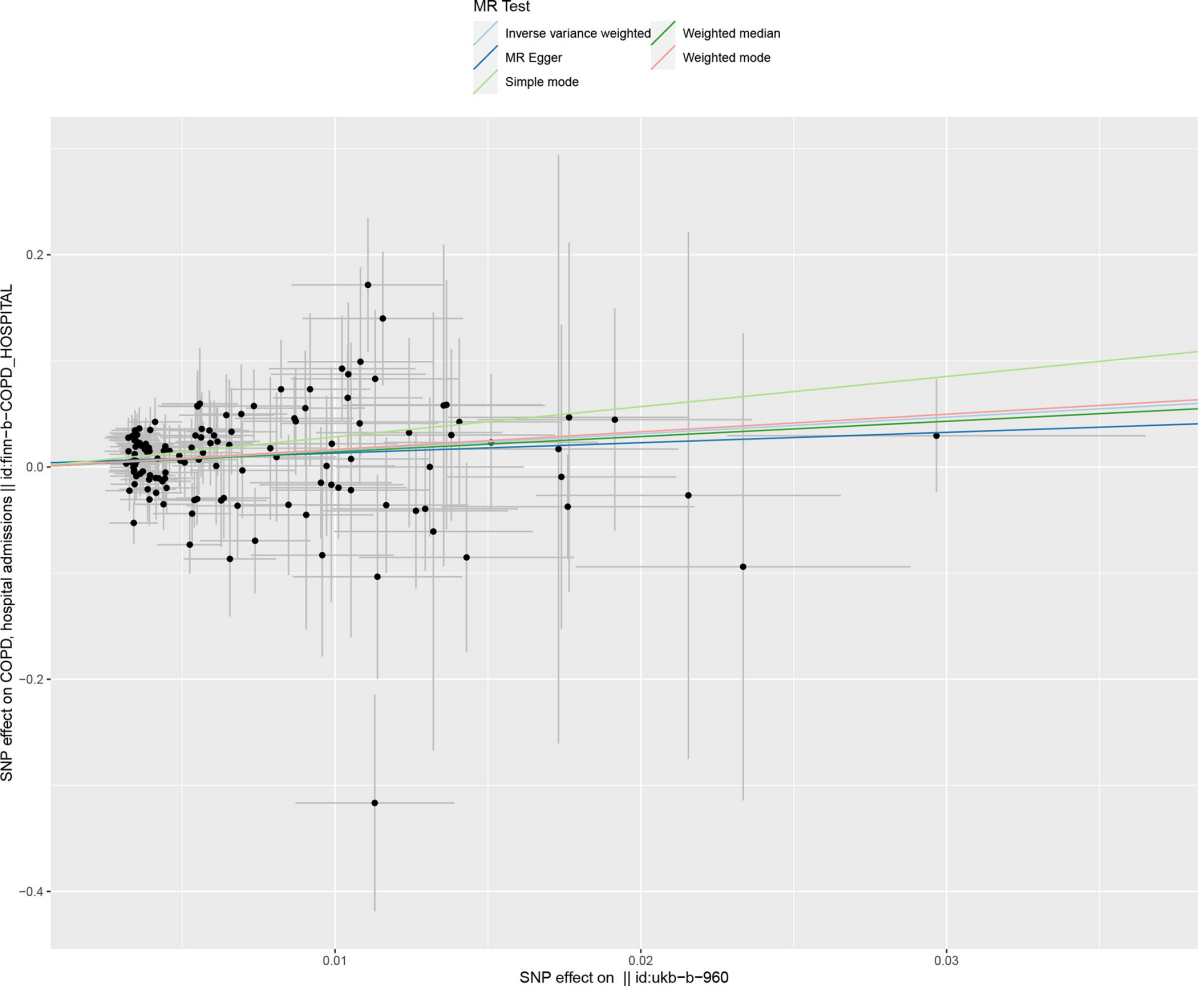
Scatter Chart of mendelian analysis between smoking/smokers in householdn and acute episodes of COPD.

**Fig 7:**
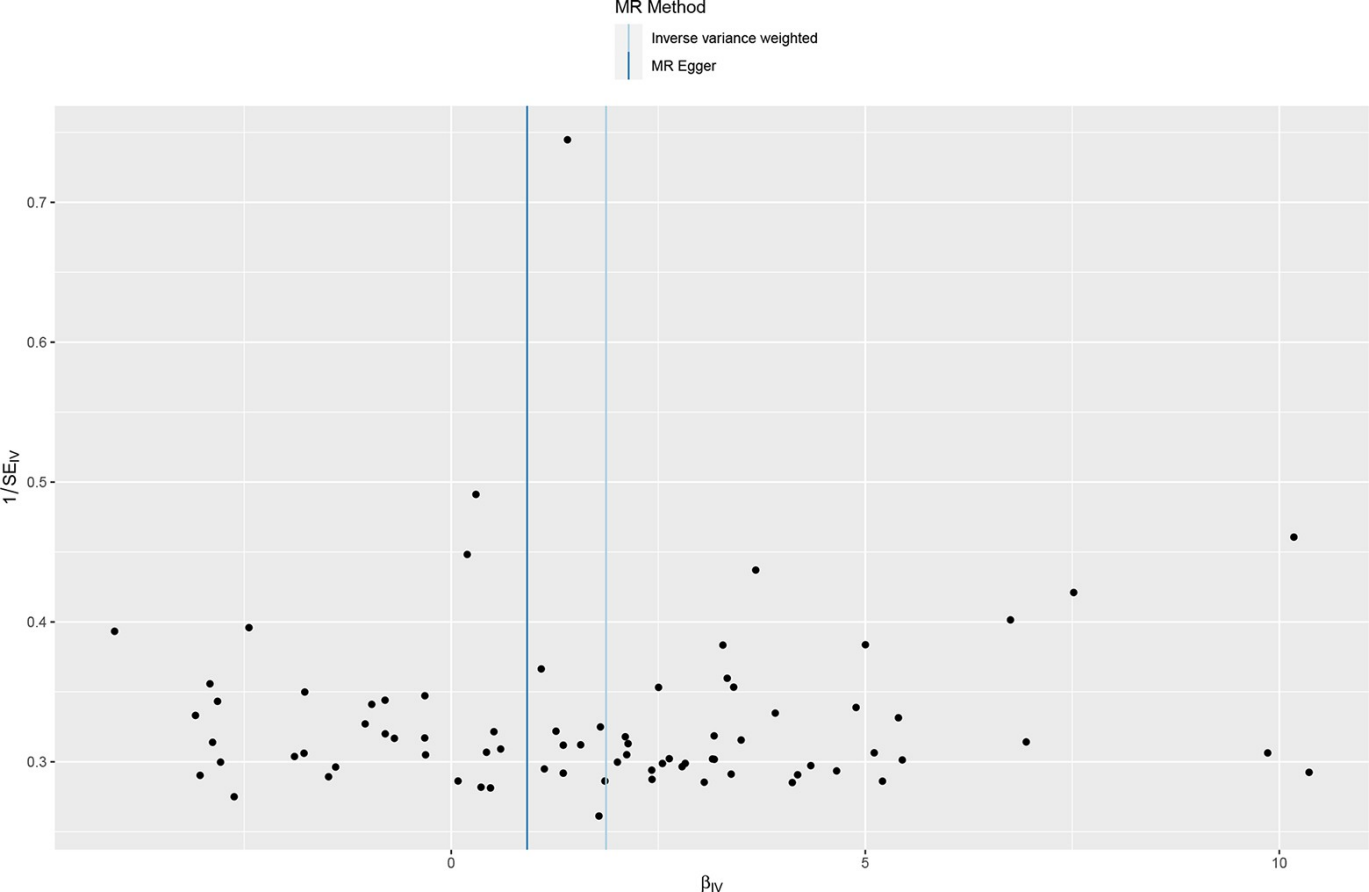
Funnel plot of mendelian analysis between ever smoking and acute episodes of COPD.

**Fig 8:**
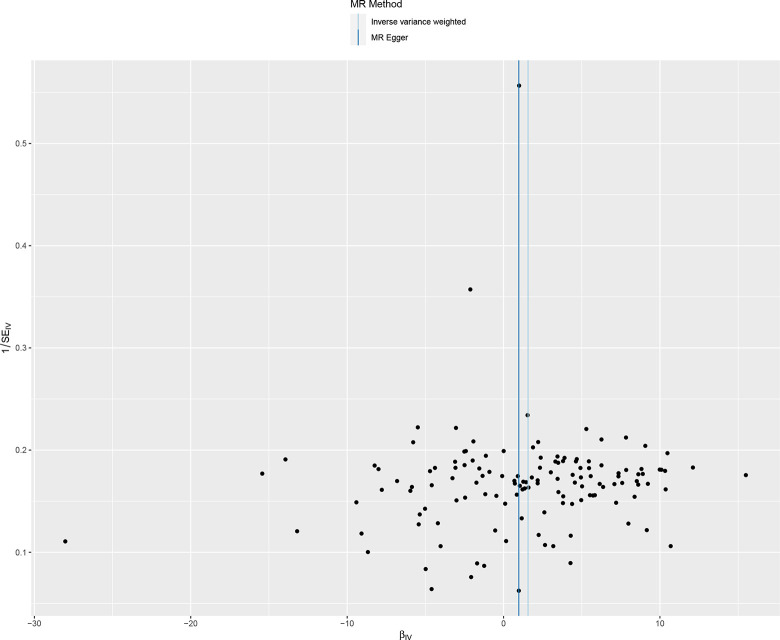
Funnel plot of mendelian analysis between smoking/smokers in householdn and acute episodes of COPD.

**Fig 9:**
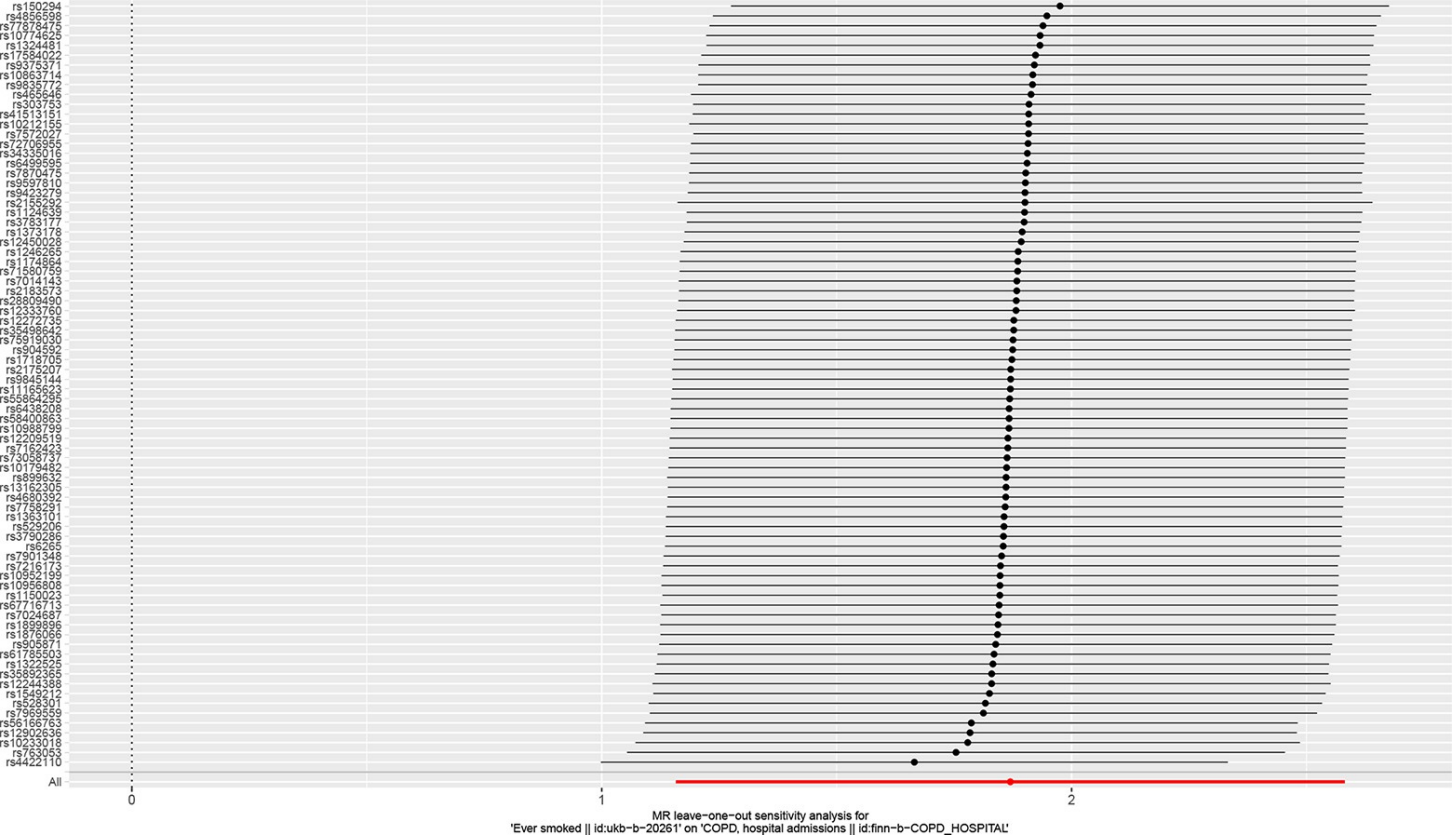
Leave one analysis of mendelian analysis ever smoking and acute episodes of COPD.

**Fig 10:**
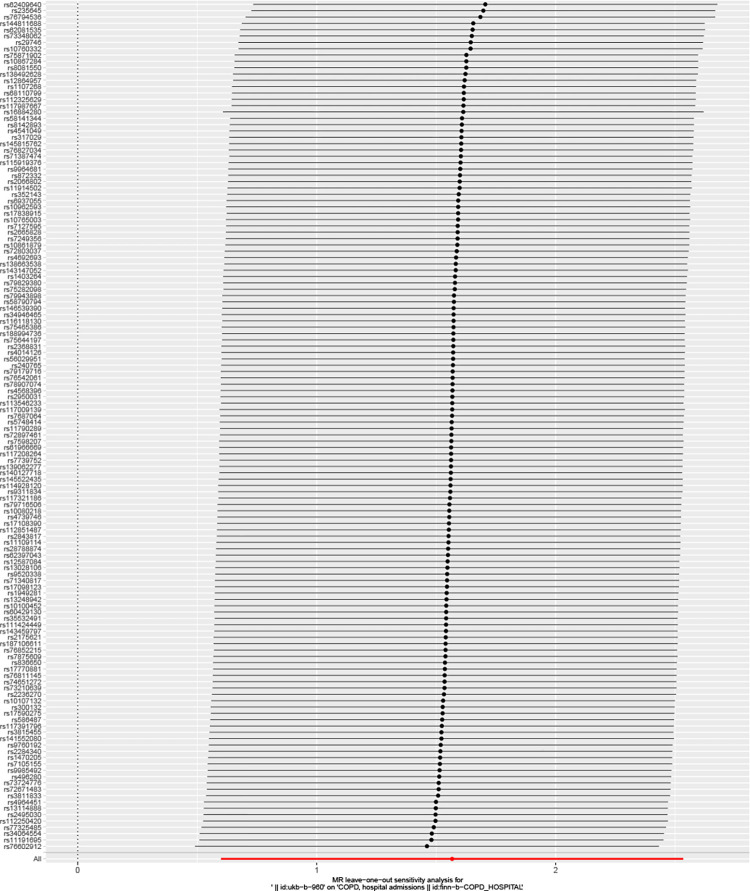
Leave one analysis of mendelian analysis between smoking/smokers in householdn and acute episodes of COPD.

We included 78 ever smoked-related SNPs ((P<5×10–8,r2<0.001,genetic distance = 10 000 KB) and 134 smoking/smokers in household-related SNPSs (P<510–5,r2<0.001, genetic distance = 10 000 KB). The IVM approach showed that ever smoke and smoking/smokers in household were associated with an increased risk of acute hospital admission with COPD and were risk factors for acute episodes of COPD. See in S4 Table. The scatter plot showed the estimated effect size of SNPs for ever smoke and smoking/smokers in household on COPD acute episode admissions (Figs 5 and 6). Heterogeneity tests showed heterogeneity among SNP effect estimates. See in S5 Table. The MR-Egger regression test did not support any evidence of directional variability. See in S5 Table. The symmetry of the funnel plot showed the same results (Figs 7 and 8). MR-PRESSO did not detect outliers. See in S5 Table. Leaveoneout plots showed no substantial change in genetically predicted risk of admission to hospital for acute COPD episodes (Figs 9 and 10). The IVM method showed that exposure to tobacco smoke at home was not associated with hospital admission for acute episodes of COPD. Sensitivity analysis and heterogeneity analysis of exposure to tobacco smoke at home are shown in S5 Table.

## Discussion

In this study, smoking/smokers in household didn”t increas the risk of COPD, while ever smoke increased the risk of COPD. Besides, ever smoke and smoking/smokers in household increased the risk of COPD acute hospital admission.

COPD is considered a leading cause of death worldwide [7]. Smoking, environmental pollution, and occupational pollutants are considered risk factors for COPD(8). When the human airways are continuously exposed to environmental pollutants, the deterioration of lung disease will be accelerated. Repeated prolonged exposure to smoke activates reactive oxygen species, symptomatic oxidative stress, and apoptosis, leading to enlargement of alveolar space and the progression of COPD [8]. We found that ever smoke increased the risk of COPD. In a prospective study, ever smoke COPD patients had declined lung function, decreased DLCO/Va, and increased frequency of emphysema on CT scan compared to never smoke COPD patients [9]. However, the mechanism by which smoke causes COPD is unknown. Studies have shown that the long-form OPA1 isotype and related proteins play a crucial role in cigarette smoke-induced lung injury, leading to mitochondrial autophagy/mitochondrial dysfunction in COPD [10]. In a meta-analysis, a history of tuberculosis/rheumatoid arthritis, exposure to biomass fuels, smoking and secondhand smoke were risk factors for COPD [11]. However, we found that exposure to tobacco smoke at home was not associated with susceptibility to COPD. We took into account the possible influence of stratification factors such as gender and age. In a large study, exposure to secondhand smoke at home and at work for more than 10 years during adulthood was associated with a 9% and 12% increase in prevalence, respectively [10]. And exposure to secondhand smoke at home during childhood was not a factor in COPD [11]. In an evaluation of 880 ever smokers with normal lung function (mean age 61 years; 52% female), using a CAT cut point of 10 or greater, we classified 51.8% as having severe symptoms, of which 15.3% experienced at least 1 exacerbation during the 1-year follow-up period [12]. Our study found that ever smoke and smoking/smokers in household were associated with an increased risk of acute COPD admission and were risk factors for acute episodes of COPD. Studies have found that patients with COPD who quit smoking have significantly lower mortality rates and use of health services (hospitalization and emergency department visits compared to continuing smokers, and patients with COPD who quit smoking have significantly better health outcomes than continuing smokers [13]. The above highlights the importance of effective smoking cessation. However, compared to smokers with no history of COPD, COPD among smokers was associated with a stronger dependence on tobacco and a lower self-control to quit smoking.

Mendelian analyses, which mostly use aggregated data from large-scale GWAS, can increase the credibility of the inferred causal relationship. A variety of MR methods and sensitivity analyses were used to enhance the credibility of the findings. However, this study has some limitations: since the GWAS pooled-level data are from European populations, the inferred causal relationships may not apply to other ethnic groups.

## Conclusion

This study found that ever smoke increased the risk of COPD by Mendelian randomization study method analysis, and that smoking/smokers in household didn”t increas the risk of COPD, while ever smoke increased the risk of COPD. We should not only strengthen the prevention propaganda for high-risk groups to reduce individual incidence, but also strengthen the management of non-pharmacological prescriptions for patients with chronic obstructive pulmonary disease to reduce the risk of acute exacerbation. This will not only effectively improve reduce the prevalence, improve the quality of life of patients and reduce the burden of medical care. In addition, due to genetic differences between ethnic groups, countries and regions, further research is needed on different populations.

## Supporting information

S1 Table Description of exposure and ending variables. (DOCX)

S2 Table Mendelian analysis of smoke in patients with COPD diagonis by doctor. (DOCX)

S3 Table Sensitivity analysis of the causal effect of smoke in patients with COPD diagonis by doctor. (DOCX)

S4 Table Mendelian analysis of smoke in patients with AECOPD. (DOCX)

S5 Table Sensitivity analysis of the causal effect of smoke in patients with AECOPD. (DOCX)

S1 File Forest plot of Mendelian analysis of ever smoke and COPD prevalence. (PDF)

S2 File Forest plot of Mendelian analysis of ever smoke and AECOPD. (PDF)

S3 File Forest plot of Mendelian analysis of exposure to tobacco at home and COPD prevalence. (PDF)

S4 File Forest plot of Mendelian analysis of exposure to tobacco at home and AECOPD. (PDF)

S5 File Forest plot of Mendelian analysis of smoking/smokers in household and COPD prevalence. (PDF)

S6 File Forest plot of Mendelian analysis of smoking/smokers in household and AECOPD. (PDF)

S7 File (ZIP)

## References

[1] GBD 2019 Chronic Respiratory Diseases Collaborators. Global burden of chronic respiratory diseases and risk factors, 1990–2019: an update from the Global Burden of Disease Study 2019. EClinicalMedicine. 2023;59:101936. doi: 10.1016/j.eclinm.2023.101936 37229504 PMC7614570

[2] Negewo NA, Gibson PG, McDonald VM. COPD and its comorbidities: Impact, measurement and mechanisms. Respirology. 2015;20(8):1160–1171. doi: 10.1111/resp.12642 26374280

[3] López-Campos JL, Tan W, Soriano JB. Global burden of COPD. Respirology. 2016;21(1):14–23. doi: 10.1111/resp.12660 26494423

[4] Agustí A, Vogelmeier C, Faner R. COPD 2020: changes and challenges. Am J Physiol Lung Cell Mol Physiol. 2020;319(5):L879–L883. doi: 10.1152/ajplung.00429.2020 32964724

[5] Lareau SC, Fahy B, Meek P, Wang A. Chronic Obstructive Pulmonary Disease (COPD). Am J Respir Crit Care Med. 2019;199(1):P1–P2. doi: 10.1164/rccm.1991P1 30592446

[6] Davey Smith G, Hemani G. Mendelian randomization: genetic anchors for causal inference in epidemiological studies. Hum Mol Genet. 2014;23(R1):R89–R98. doi: 10.1093/hmg/ddu328 25064373 PMC4170722

[7] Ko FW, Chan KP, Hui DS, Goddard JR, Shaw JG, Reid DW, et al. Acute exacerbation of COPD. Respirology. 2016;21(7):1152–1165. doi: 10.1111/resp.12780 27028990 PMC7169165

[8] Vij N, Chandramani-Shivalingappa P, Van Westphal C, Hole R, Bodas M. Cigarette smoke-induced autophagy impairment accelerates lung aging, COPD-emphysema exacerbations and pathogenesis. Am J Physiol Cell Physiol. 2018;314(1):C73–C87. doi: 10.1152/ajpcell.00110.2016 27413169 PMC5866380

[9] Tan WC, Sin DD, Bourbeau J, Hernandez P, Chapman KR, Cowie R, et al. Characteristics of COPD in never-smokers and ever-smokers in the general population: results from the CanCOLD study. Thorax. 2015;70(9):822–829. doi: 10.1136/thoraxjnl-2015-206938 26048404

[10] Maremanda KP, Sundar IK, Rahman I. Role of inner mitochondrial protein OPA1 in mitochondrial dysfunction by tobacco smoking and in the pathogenesis of COPD. Redox Biol. 2021;45:102055. doi: 10.1016/j.redox.2021.102055 34214709 PMC8258692

[11] van Koeverden I, Blanc PD, Bowler RP, Arjomandi M. Secondhand tobacco smoke and COPD risk in smokers: A COPDGene study cohort subgroup analysis. Copd. 2015;12(2):182–189. doi: 10.3109/15412555.2014.922173 24983136 PMC4820340

[12] Martinez CH, Murray S, Barr RG, Bleecker E, Bowler RP, Christenson SA, et al. Respiratory symptoms items from the COPD assessment test identify ever-smokers with preserved lung function at higher risk for poor respiratory outcomes. an analysis of the subpopulations and intermediate outcome measures in COPD study cohort. Ann Am Thorac Soc. 2017;14(5):636–642. doi: 10.1513/AnnalsATS.201610-815OC 28459622 PMC5427740

[13] Tashkin DP. Smoking cessation in chronic obstructive pulmonary disease. Semin Respir Crit Care Med. 2015;36(4):491–507. doi: 10.1055/s-0035-1555610 26238637

