# Supplementary material for: Application of mendelian randomization to study the causal relationship between smoking and the risk of chronic obstructive pulmonary disease
Source: PLoS One. 2023 Jul 28;18(7):e0288783. doi: 10.1371/journal.pone.0288783 (PMC10381044; doi:10.1371/journal.pone.0288783)
Supplement: S1 Table — (DOCX) [file pone.0288783.s001.docx]

| Variable | ID | Sample size | SNP | Race | Gender | Year |
| --- | --- | --- | --- | --- | --- | --- |
| Ever smoked | ukb-b-20261 | 461,066 | 9,851,867 | European | Males and Females | 2018 |
| Smoking/smokers in household | ukb-b-960 | 425,516 | 9,851,867 | European | Males and Females | 2018 |
| Exposure to tobacco smoke at home | ukb-b-4462 | 417,693 | 9,851,867 | European | Males and Females | 2018 |
| COPD, hospital admissions | finn-b-COPD_HOSPITAL | 218,792 | 16,380,466 | European | Males and Females | 2021 |
| Doctor diagnosed COPD (chronic obstructive pulmonary disease) | ukb-b-20464 | 112,583 | 9,851,867 | European | Males and Females | 2018 |

Table S1 Description of exposure and ending variables
