# Supplementary material for: Application of mendelian randomization to study the causal relationship between smoking and the risk of chronic obstructive pulmonary disease
Source: PLoS One. 2023 Jul 28;18(7):e0288783. doi: 10.1371/journal.pone.0288783 (PMC10381044; doi:10.1371/journal.pone.0288783)
Supplement: S2 Table — (DOCX) [file pone.0288783.s002.docx]

Table S2 Mendelian analysis of smoke in patients with COPD diagonis by doctor

|  | **MR Egger** |  | **Weighted median** |  | **Inverse variance weighted** |  | **Simple mode** |  | **Weighted mode** |  |
| --- | --- | --- | --- | --- | --- | --- | --- | --- | --- | --- |
|  | SE | P | SE | P | SE | P | SE | P | SE | P |
| ever smoked | 0.057 | 0.052 | 0.014 | 0.004 | 0.011 | 0.000 | 0.039 | 0.496 | 0.046 | 0.816 |
| exposure to tobacco smoke at home | 2.826 | 0.697 | 1.396 | 0.716 | 0.983 | 0.903 | 2.952 | 0.225 | 2.787 | 0.207 |
| smoking/smokersin in household | 0.292 | 0.472 | 0.031 | 0.695 | 0.023 | 0.997 | 0.069 | 0.642 | 0.072 | 0.722 |
