# Supplementary material for: Application of mendelian randomization to study the causal relationship between smoking and the risk of chronic obstructive pulmonary disease
Source: PLoS One. 2023 Jul 28;18(7):e0288783. doi: 10.1371/journal.pone.0288783 (PMC10381044; doi:10.1371/journal.pone.0288783)
Supplement: S3 Table — (DOCX) [file pone.0288783.s003.docx]

|  | Heterogeneity test | | | | Multiple validity test | | | Mppresso | | | |
| --- | --- | --- | --- | --- | --- | --- | --- | --- | --- | --- | --- |
|  | MR Egger | | Inverse variance weighted | |  | | | beta.exposure | | beta.exposure Outlier-corrected | |
|  | Q | P | Q | P | Egger intercept | SE | P | Estimate | P | Estimate | P |
| ever smoked | 80.869 | 0.103 | 82.982 | 0.090 | 0.000 | 0.000 | 0.194 | 0.035 | 0.001 | NA | NA |
| exposure to tobacco smoke at home | 55.895 | 0.175 | 56.524 | 0.187 | -0.001 | 0.001 | 0.471 | 0.006 | 0.798 | NA | NA |
| smoking/smokers in household | 24.240 | 0.335 | 24.480 | 0.378 | 0.008 | 0.016 | 0.645 | 0.967 | 0.387 | NA | NA |

Table S3 Sensitivity analysis of the causal effect of smoke in patients with COPD diagonis by doctor
