# Supplementary material for: Application of mendelian randomization to study the causal relationship between smoking and the risk of chronic obstructive pulmonary disease
Source: PLoS One. 2023 Jul 28;18(7):e0288783. doi: 10.1371/journal.pone.0288783 (PMC10381044; doi:10.1371/journal.pone.0288783)
Supplement: S4 Table — (DOCX) [file pone.0288783.s004.docx]

Table S4 Mendelian analysis of smoke in patients with AECOPD

|  | **MR Egger** |  | **Weighted median** |  | **Inverse variance weighted** |  | **Simple mode** |  | **Weighted mode** |  |
| --- | --- | --- | --- | --- | --- | --- | --- | --- | --- | --- |
|  | SE | P | SE | P | SE | P | SE | P | SE | P |
| ever smoked | 1.761 | 0.604 | 0.513 | 0.004 | 0.363 | 0.000 | 1.207 | 0.056 | 1.056 | 0.098 |
| exposure to tobacco smoke at home | 2.763 | 0.934 | 1.328 | 0.830 | 0.977 | 0.823 | 2.943 | 0.496 | 1.611 | 0.425 |
| smoking/smokers in household | 1.091 | 0.372 | 0.874 | 0.100 | 0.493 | 0.002 | 2.688 | 0.595 | 1.422 | 0.245 |
