# Supplementary material for: Application of mendelian randomization to study the causal relationship between smoking and the risk of chronic obstructive pulmonary disease
Source: PLoS One. 2023 Jul 28;18(7):e0288783. doi: 10.1371/journal.pone.0288783 (PMC10381044; doi:10.1371/journal.pone.0288783)
Supplement: S5 Table — (DOCX) [file pone.0288783.s005.docx]

Table S5 Sensitivity analysis of the causal effect of smoke in patients with AECOPD

|  | Heterogeneity test | | | | Multiple validity test | | | Mppresso |  |  |  |
| --- | --- | --- | --- | --- | --- | --- | --- | --- | --- | --- | --- |
|  | MR Egger |  | Inverse variance weighted |  |  |  |  | beta.exposure |  | beta.exposure Outlier-corrected |  |
|  | Q | P | Q | P | Egger intercept | SE | P | Estimate | P | Estimate | P |
| ever smoked | 89.421 | 0.139 | 89.781 | 0.151 | 0.007 | 0.012 | 0.582 | 1.735 | 0.000 | NA | NA |
| exposure to tobacco smoke at home | 164.190 | 0.001 | 165.808 | 0.001 | -0.008 | 0.008 | 0.298 | 1.463 | 0.293 | NA | NA |
| smoking/smokers in household | 26.887 | 0.138 | 27.399 | 0.158 | -0.014 | 0.023 | 0.544 | 0.035 | 0.001 | NA | NA |
