# Supplementary figures and images for: Application of mendelian randomization to study the causal relationship between smoking and the risk of chronic obstructive pulmonary disease
Source: PLoS One. 2023 Jul 28;18(7):e0288783. doi: 10.1371/journal.pone.0288783 (PMC10381044; doi:10.1371/journal.pone.0288783)

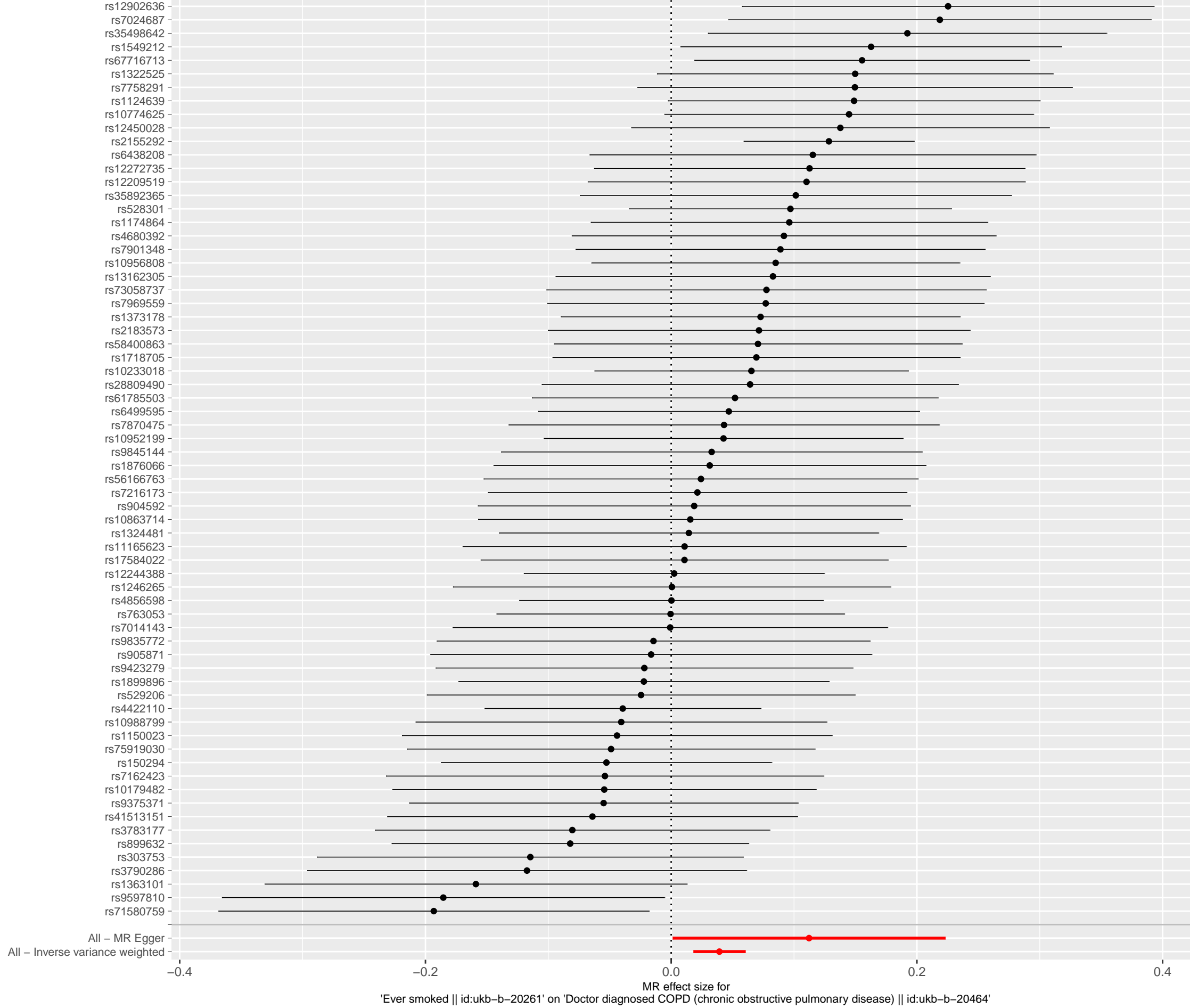

Supplement: S1 File — (PDF) [file pone.0288783.s006.pdf]

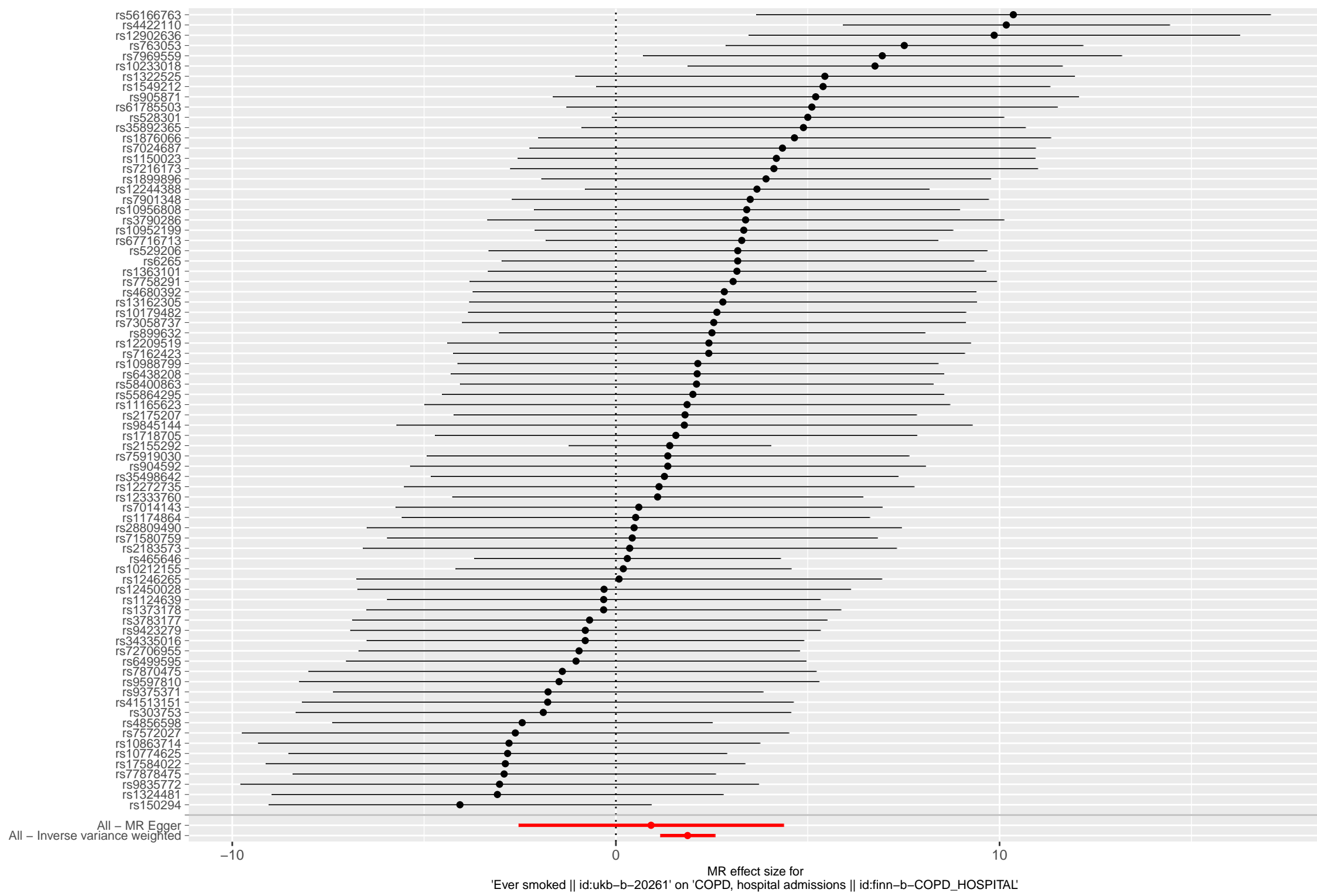

Supplement: S2 File — (PDF) [file pone.0288783.s007.pdf]

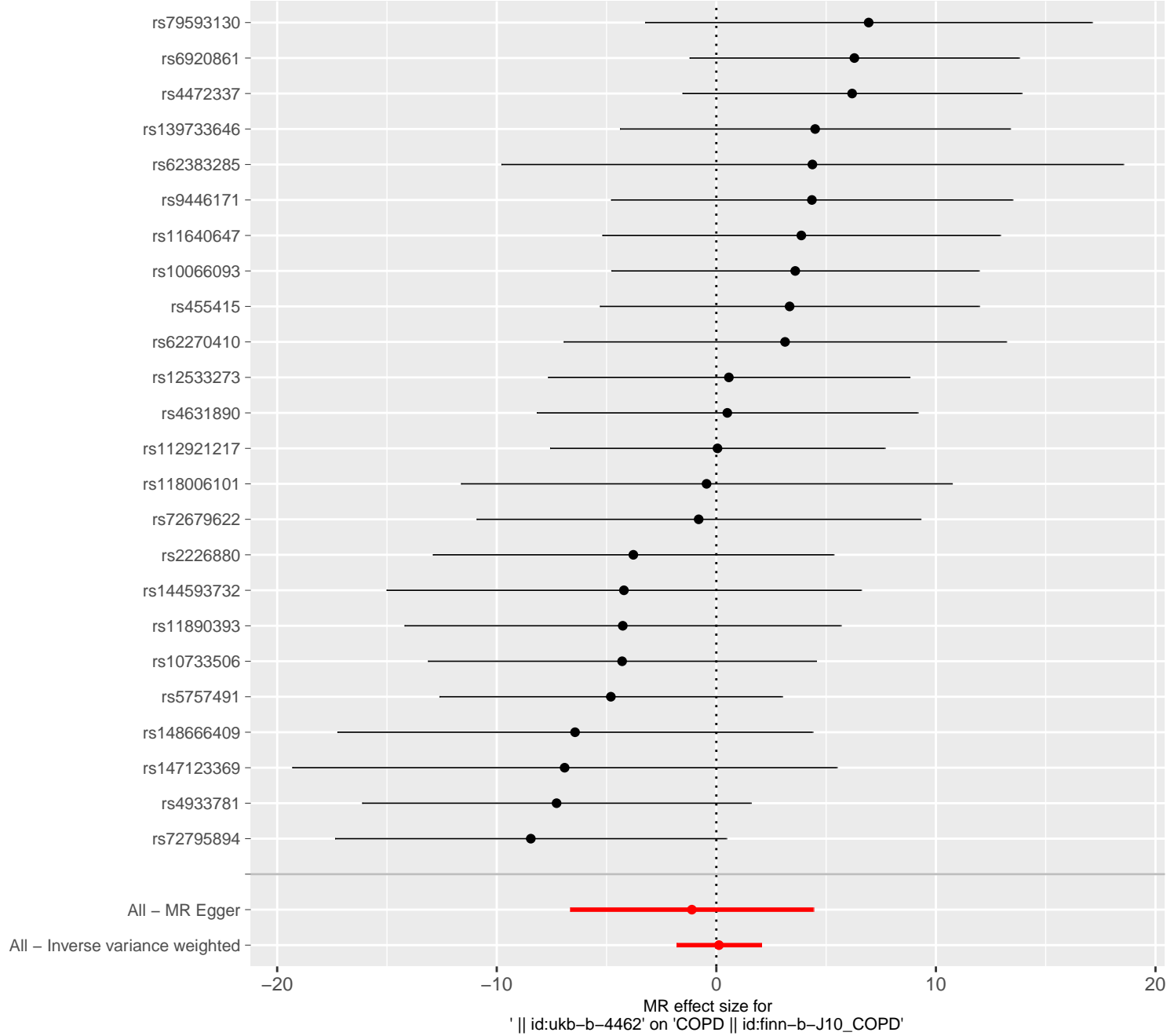

Supplement: S3 File — (PDF) [file pone.0288783.s008.pdf]

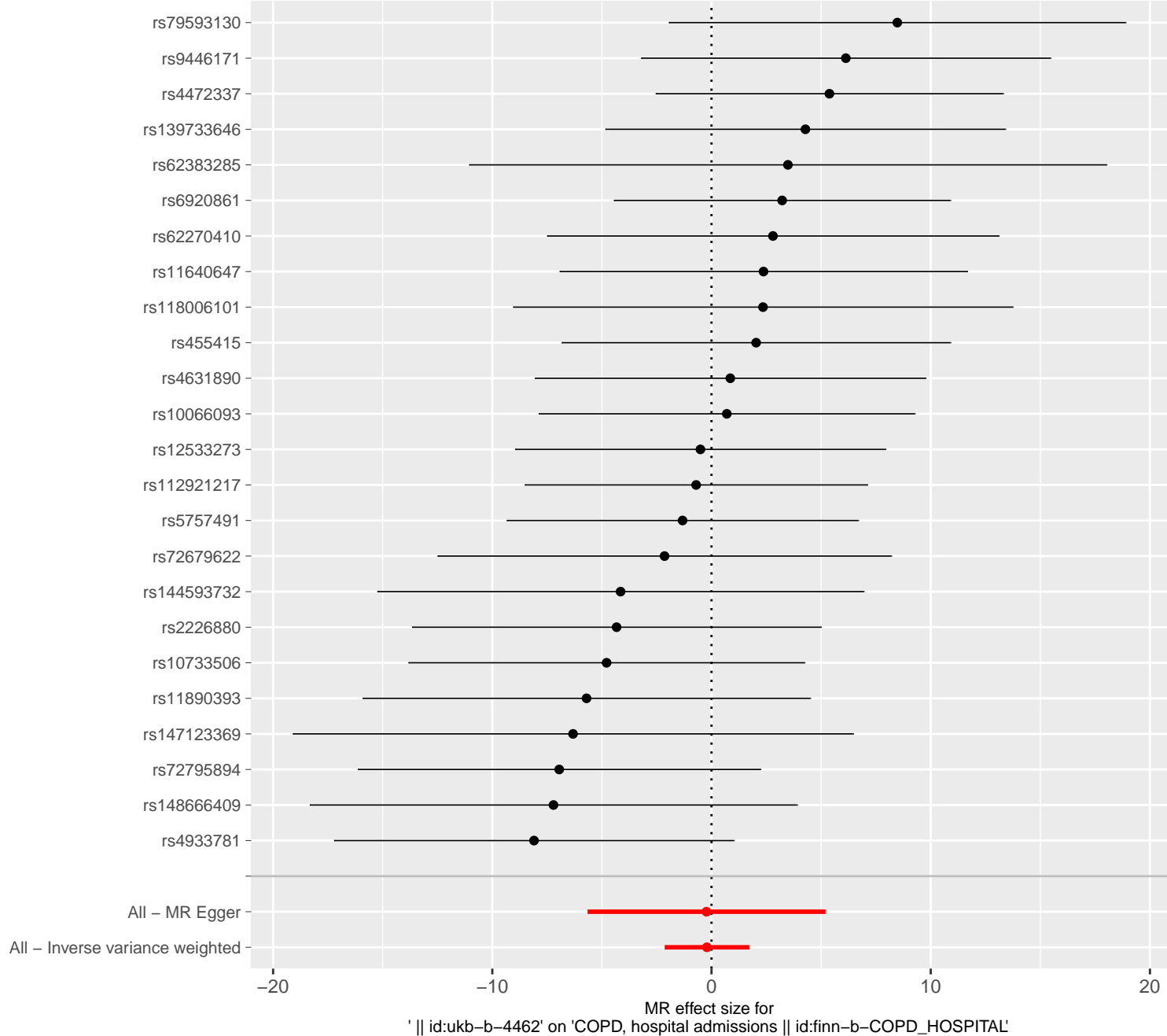

Supplement: S4 File — (PDF) [file pone.0288783.s009.pdf]

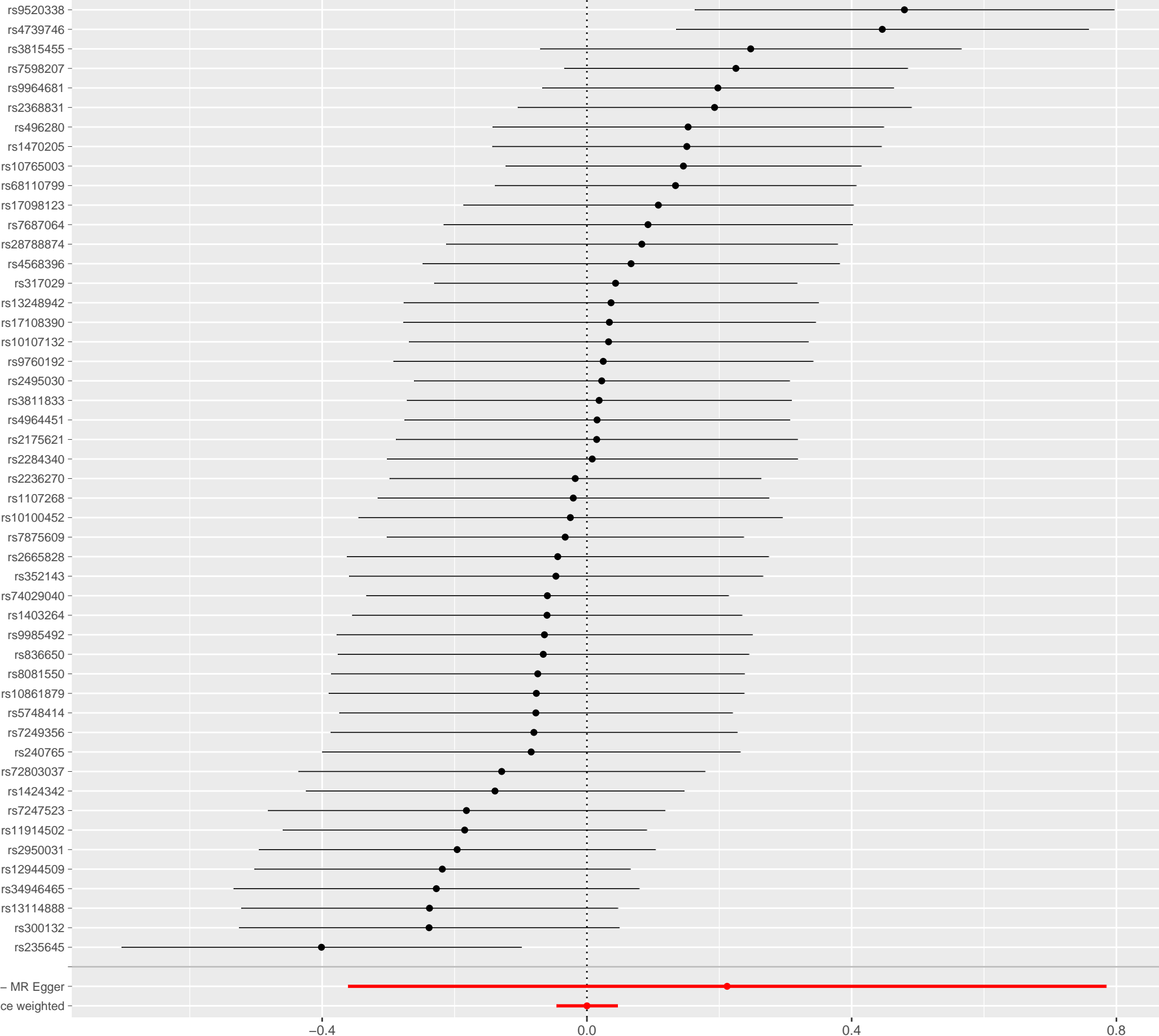

' || id:ukb-b-960' on 'Doctor diagnosed COPD (chronic obstructive pulmonary disease)' || id:ukb-b-20464'

Supplement: S5 File — (PDF) [file pone.0288783.s010.pdf]

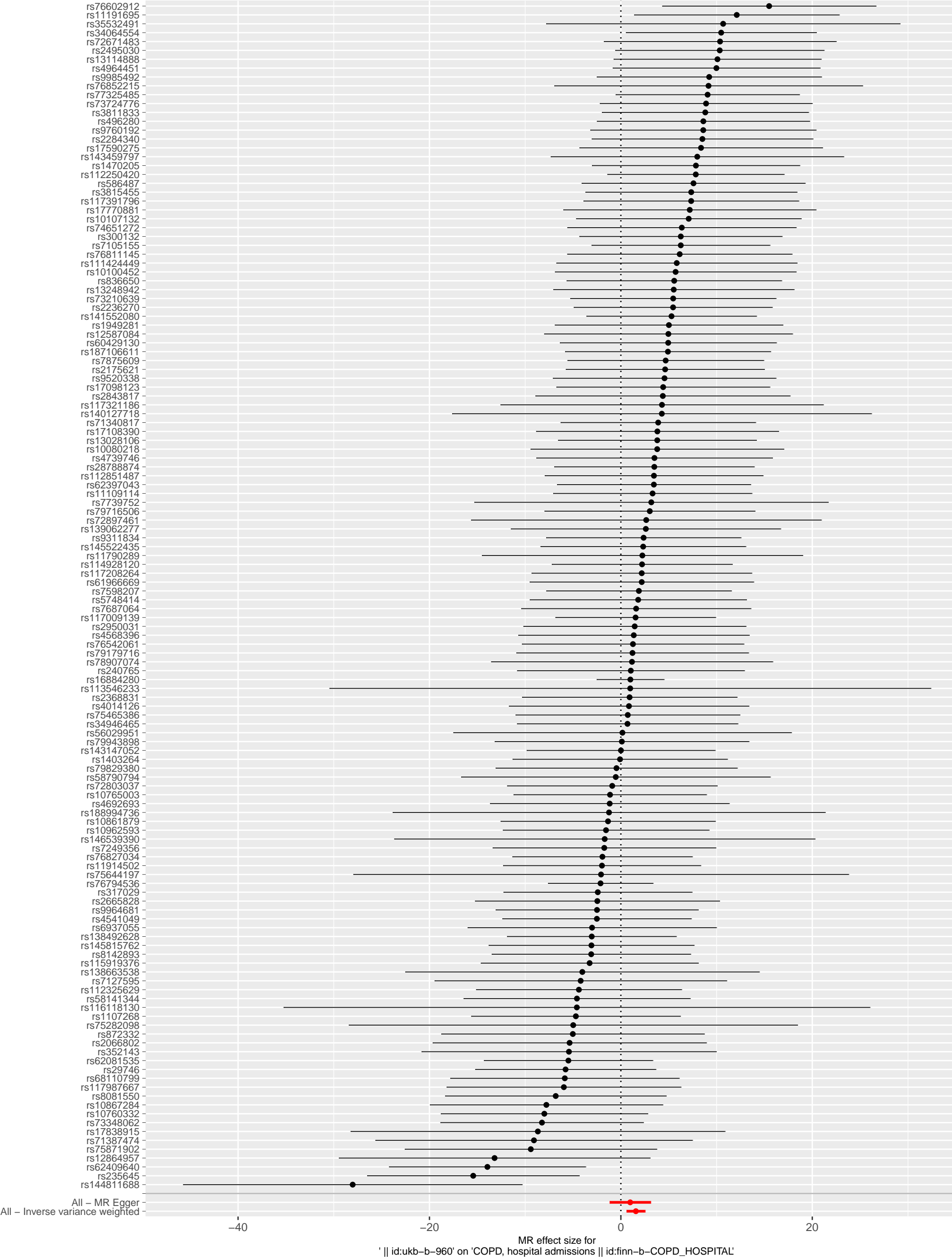

Supplement: S6 File — (PDF) [file pone.0288783.s011.pdf]
